# The temperature-dependent expression of type II secretion system controls extracellular product secretion and virulence in mesophilic *Aeromonas salmonida* SRW-OG1

**DOI:** 10.3389/fcimb.2022.945000

**Published:** 2022-08-01

**Authors:** Xin Yi, Yunong Chen, Hongyan Cai, Jiajia Wang, Youyu Zhang, ZhiQin Zhu, Mao Lin, Yingxue Qin, XingLong Jiang, Xiaojin Xu

**Affiliations:** ^1^ Fisheries College, Key Laboratory of Healthy Mariculture for the East China Sea, Ministry of Agriculture and Rural Affairs, Engineering Research Center of the Modern Technology for Eel Industry, Jimei University, Xiamen, China; ^2^ Engineering Research Center of the Modern Technology for Eel Industry, Ministry of Education, Xiamen, China; ^3^ Institute of Electromagnetics and Acoustics, School of Electronic Science and Engineering, Xiamen University, Xiamen, China

**Keywords:** *Aeromonas salmona*, mesophilic, *tatA*, *tatB*, *tatC*, virulence

## Abstract

*Aeromonas salmonicida* is a typical cold water bacterial pathogen that causes furunculosis in many freshwater and marine fish species worldwide. In our previous study, the pathogenic *A. salmonicida* (SRW-OG1) was isolated from a warm water fish, *Epinephelus coioides* was genomics and transcriptomics analyzed. Type II secretion system was found in the genome of *A. salmonicida* SRW-OG1, while the expressions of *tatA, tatB*, and *tatC* were significantly affected by temperature stress. Also, sequence alignment analysis, homology analysis and protein secondary structure function analysis showed that *tatA*, *tatB*, and *tatC* were highly conservative, indicating their biological significance. In this study, by constructing the mutants of *tatA, tatB*, and *tatC*, we investigated the mechanisms underlying temperature-dependent virulence regulation in mesophilic *A. salmonida* SRW-OG1. According to our results, *tatA*, *tatB*, and *tatC* mutants presented a distinct reduction in adhesion, hemolysis, biofilm formation and motility. Compared to wild-type strain, inhibition of the expression of *tatA*, *tatB*, and *tatC* resulted in a decrease in biofilm formation by about 23.66%, 19.63% and 40.13%, and a decrease in adhesion ability by approximately 77.69%, 80.41% and 62.14% compared with that of the wild-type strain. Furthermore, *tatA*, *tatB*, and *tatC* mutants also showed evidently reduced extracellular enzymatic activities, including amylase, protease, lipase, hemolysis and lecithinase. The genes affecting amylase, protease, lipase, hemolysis, and lecithinase of *A. salmonicida* SRW-OG1 were identified as *cyoE*, *ahhh1*, *lipA*, *lipB*, *pulA*, *HED66_RS01350, HED66_RS19960*, *aspA*, *fabD*, and *gpsA*, which were notably affected by temperature stress and mutant of *tatA, tatB*, and *tatC*. All above, *tatA, tatB* and *tatC* regulate the virulence of *A. salmonicida* SRW-OG1 by affecting biofilm formation, adhesion, and enzymatic activity of extracellular products, and are simultaneously engaged in temperature-dependent pathogenicity.

## Introduction

*Aeromonas salmonicida*, which is distributed worldwide, is a psychrophilic gram-negative bacterium and is one of the few non-motile, facultatively anaerobic strains of the genus *Aeromonas.* There are five accepted subspecies of *A. salmonicida*: *A. salmonicida subsp. Salmonicida* (known as typical)*, masoucida, achromogenes, pectinolytica*, and *smithia* (Austin et al., 2007; Merino and Tomás, 2016; He et al., 2022). *A. salmonicida* has a wide range of hosts, infecting not only infecting salmon and trout (Du et al., 2015), but also *Cyprinus carpio* (Maurice and Tinman, 2000), *Anoplopoma fimbria* (Vasquez et al., 2020), *Gadusmorhua* (Soto-Dávila et al., 2019), *Scophthalmus maximus* (Xu et al., 2021), and *Percafluviatilis* (Rupp et al., 2019). The symptoms of infection are mainly “furunculosis” (skin ulcers) and ‘septicemia’ in salmons (Salomón et al., 2021) and *C. carpio* (Bhat et al., 2021). *A. salmonicida* SRW-OG1 was isolated in our laboratory from *Epinephelus coioides* suffering from furunculosis in Dongshan County, Zhangzhou City, Fujian Province (Zhong et al., 2021). Surprisingly, the pathogen was isolated at 28°C. Through temperature stress, we found that the bacterium is highly mesophilic and can grow even at 37°C. That is contrary to the conclusion of many scholars that *A. salmonicida* is a psychrophilic bacteria (Meng et al., 2017).

Temperature is a pivotal environmental factor for fish disease outbreaks. In response to temperature changes, bacteria need to adjust their physiology to cope with the stimuli and stresses brought about by environmental changes (Huntingford et al., 2007). The outbreak of several common fish diseases has an absolute relationship with water temperature: with the decrease in water temperature, the probability of cold water disease (Kobayashi et al., 2000), cold water vibrosis, saprolegnia (Sformo et al., 2021), red skin disease, and red mouth disease (Fernandez et al., 2003) will increase significantly; conversely, elevated water temperatures may lead to lactococcal disease, Edwards disease, bacterial sepsis, and carp herpes disease. Interestingly, some bacterial diseases occur at temperatures far below the temperature at which bacteria reach their fastest growth rate, known as the optimal bacterial growth temperature. The optimal growth temperature of *Escherichia coli* is 37°C, but the lethality of fish and mice is higher at 20°C (Wu et al., 2010). Studies have shown that the effect of temperature on bacterial metabolism is mainly manifested in changing the activity of enzymes. However, the temperature accommodation immune disease prevention mechanism of bacteria is not only the acceleration-deceleration regulation of enzymatic activity, but also affects the expression of genes in respond through a variety of biological functions (Guijarro et al., 2015). In the expression study of *Yersinia ruckeri* specific secretory genes, it was found that the expression level of the type IV secretory system encoded by the *traHIJKCLMN* operon at the optimal growth temperature was 64% lower than that at 18°C (Méndez and Guijarro, 2013). Similarly, the Yrp1 protease and YhlA hemolysin of *Y. ruckeri* showed three folds the gene expression at 18°C than at 28°C. To investigate the mechanism underlying the virulence regulation at different temperatures, the genomics and transcriptomics analysis on *A. salmonicida* SRW-OG1 have been carried out. Type II secretion system was found in the genome of *A. salmonicida* SRW-OG1 (Huang et al., 2020a), while the expressions of *tatA*, *tatB*, and *tatC*, which belong to Type II secretion system (T2SS), were greatly affected by temperature stress.

T2SS is a multi-protein secretion system widely present in Gram-negative bacteria and plays an essential role in pathogenic mechanisms. Most of the enzymes secreted by T2SS have degradative functions, increasing the destructive effect of bacteria on host cells and tissues. The twin-arginine translocation (Tat) system is a classic transmembrane transport system of the type II secretion system. It is an important part of the bacterial secretion system, but it is absent in Mycoplasma, Methanogens, and *Borrelia burgdorferi* (Palmer and Berks, 2012). In *Pseudomonas aeruginosa*, the Tat system mediates the first step in the secretion of the exoproteins PlcH and PlcN (Voulhoux et al., 2001). While *Salmonella* lacks the Tat system, the cell wall is destroyed, making it more sensitive to EDTA and SDS, and the morphology of the bacteria will become longer or chain-like (Stanley et al., 2001). The absence of the Tat system in *Ralstonia solanacearum* will seriously affect its physiological functions, such as a severe reduction in the ability of nitrate utilization, cell division, biofilm stabilization, and growth tendency (González et al., 2007). *Legionella pneumophila tatB* and *tatC* mutants have significantly reduced ability to form biofilms compared to wild type, resulting from a combination of outer membrane and flagella defects (Buck et al., 2006). The *P. aeruginosa tatC* mutant also showed a conspicuously reduced biofilm formation ability due to the weakened bacterial motility. However, the relationship between the Tat system of many pathogenic bacteria and the ability to form biofilms has not been studied. Moreover, some pathogens have proved that the Tat system has no obvious relationship with the formation of biofilms, such as *Agrobacterium tumefaciens*, etc. (Ding and Christie, 2003). In a rat model to simulate chronic lung infection with *P. aeruginosa*, it was found that *tatC* mutants failed to cause lung damage, indicating that the Tat system plays a crucial role in the regulation of bacterial virulence factors (Ochsner et al., 2002). Our previous transcriptomics analysis speculated that the Tat system was closely related to the temperature-dependent regulation in *A. salmonicida* SRW-OG1.

The genes (*tatA*, *tatB*, and *tatC*) knockout strains of *A. salmonicida* were constructed in our studies. Meanwhile, we extracted extracellular products at different temperatures and used enzyme activity plates and bioinformatics analysis to identify genes, and the expression was affected by temperature. It was found that 18°C, 28°C, and 37°C played various regulatory roles in extracellular proteases (ECP) production and movement. The band with a molecular weight of 35KDa was an ordinary band of ECP extracted at three different temperatures. We further elucidated the virulence regulation mechanism of the Tat system through various physiological changes and direct regulation of the expression of synthetase or secretase encoding genes. To determine how these genes regulate adhesion and biofilm formation under natural conditions and thus affect protein output. It is helpful to understand further the role of the secretion system in the pathogenesis of *A. salmonicida*, and provide new targets and ideas for the treatment and prevention of *A. salmonicida*.

## Materials and methods

### Bacterial strains and culture conditions

*A. salmonicida* (SRW-OG1) was isolated from naturally infected *Epinephelus coioides* in our laboratory (Huang et al., 2020a). After artificial infection, the strain was identified as a pathogenic strain and confirmed as *A. salmonicida* by biochemical identification and 16S rRNA sequencing. It was stored at -80°C in the refrigerator. *A. salmonicida* were grown in LB broth or agar at 18°C (pH=7, 2% NaCl, 220 r.p.m.). The pKD46 plasmid was purchased from the BioVector plasmid carrier strain cell gene storage center, and we previously modified it and replaced the Amp resistance gene with the Cm resistance gene to obtain the pKD46-Cm plasmid. *Escherichia coli* containing pKD46-Cm plasmid was cultured in LB broth or agar at 37°C. *E. coli* containing pACYC184 plasmid was stored in our laboratory and cultured in LB broth containing 1% (w/v) NaCl and appropriate antibiotics at 37°C. Antibiotics used were 50 μg/ml tetracycline (Tet) and 34 μg/ml chloramphenicol (Cm) (Holden et al., 2001).

### Construction of *tatA*, *tatB*, *tatC* mutants of *A. salmonicida*

Based on the *A. salmonicida tatA*, *tatB*, and *tatC* gene sequences of *A. salmonicida*, primers with homologous arms were designed with SnapGene and synthesized (primer sequences were shown in **Table S1**). The 5’ termini of the primers were homologous to the 10-bp upstream and downstream flanking regions of the knocked-out gene. The 3’ termini of the primers were homologous to the end of the Tet resistance gene. PCR amplification was performed using 2×Pfu PCR MasterMix kit. After PCR amplification, the target fragments (with Tet resistance) of *tatA*, *tatB*, and *tatC* were respectively constructed. Plasmid pKD46-Cm was transformed into *A. salmonicida* by electroporation and cultured to OD_600_ = 0.3. After adding 30 mmol/L L-arabinose, the recombinant enzymes Exo, Bet, and Gam of pKD46-Cm were fully expressed. The targeting fragments were then transformed into *A. salmonicida* by electroporation. Positive clones were screened with Tet, and positive colonies were selected for PCR analysis and gene sequencing verification (Murphy, 1998; Datsenko and Wanner, 2000). Primers used for PCR amplification and sequencing were shown in **Table S2**. In the same way, Δ*tatB* and Δ*tatC* mutants were constructed from wild-type *A. salmonicida*.

### qRT-PCR

qRT-PCR was performed using a QuantStudio 6 Flex real-time PCR system (Life Technologies Inc., Carlsbad, CA, U.S.A) (Rodriguez et al., 2013). The 16S rRNA gene was selected as the reference gene (primer sequences were shown in **Table S3**). Each group was subjected to 3 biological replicates. The relative expression level of genes was calculated with the 2^−ΔΔCt^ method (Zuo et al., 2019; Huang et al., 2020b).

### Growth curve test

According to the previous description (He et al., 2022), we adjusted the concentration of bacterial solution to OD_600 =_ 0.1, then took 10 μL of bacterial suspension and 190 μL of sterile LB liquid medium, mixed them, and dispensed into 96-well cell culture plates. Eight parallel experiments were set up for each strain. The 96-well cell culture plate was placed in a 28°C incubator, and the OD_600_ was measured and recorded every half an hour until the stable growth phase was reached, and the growth curve was drawn according to the obtained results.

### Soft agar plate exercise test

According to the previous description (Qi et al., 2022), the concentration of the bacterial solution from wild type and three mutant strains was adjusted to OD_600 =_ 0.2, and 1μL of the bacterial suspension was taken to measure the motility of *A. salmonicida* by the semi-solid agar method. Colony diameters were measured after overnight incubation at 28°C (Li et al., 2022).

### Biofilm formation test

The bacteria were cultured on LB overnight and then suspended in 0.01M phosphate buffered saline (PBS, pH = 7.2). The bacterial suspension was adjusted to OD_600_ = 0.2 (2.0×10^8^ CFU/mL) in 0.01M PBS (pH = 7.2). 200 μL suspension was added to 96-well microporous plate (polystyrene). Biofilm production was analyzed by incubating 96-well cell culture plates with 0.1% crystal violet solution (Merck KGaA, Germany) for 15 minutes as previously described (Xu et al., 2022). The stained biofilm was recorded with a multifunctional microplate detector after dissolving 200μl of 33% acetic acid measured by OD_590_ nm.

### Hemolysis test

Hemolysis analysis was performed as previously described (He et al., 2020). We adjusted the bacterial solution to the same concentration, and used a multifunctional microplate detector to record the OD_540_ nm to detect the released hemoglobin. The total hemolysis rate was calculated by comparing the OD_540_ nm of the negative control (PBS) and positive (ddH2O) samples, and eight parallel experiments were set up for each strain.

### *In Vitro* adhesion test

Bacterial adhesion assays were performed as previously described (Huang et al., 2021a). 20 μL mucus of *E. coioides* was evenly added onto a 22 mm × 22 mm glass slide, then placed overnight, and fixed with methanol for 20 minutes at room temperature. The bacterial suspension was adjusted to a final concentration of OD_600 =_ 0.2 (2.0×10^8^ CFU/mL) with PBS. 200 μl bacterial suspension was spread evenly on the glass slide containing mucus, incubated at 28°C for 2 hours, and washed 4 times with PBS (Li et al., 2019). The bacteria were fixed in 4% methanol for 30 minutes and stained with 0.1% crystal violet for 3 minutes. The slides were observed under a light microscope (×1000), and 15 microscope fields were selected for bacterial counts. Sterile PBS was used as a negative control. Three trials were performed for each group.

### Preparation of extracellular products

According to the description by (Austin and Rodgers, 1980; Zhang and Austin, 2000), the extracellular products of *A*. *salmonicida* cultured at different temperatures were prepared by glass paper-covered plate technology. Briefly, 0.2ml overnight culture (OD600 = 0.4) was applied to each TSA plate covered with sterile glass paper. After incubation at 28°C for 48 h, the cover was transferred to the empty culture dish cover. The bacterial cells were scraped in 4.0 ml phosphate buffered saline (PBS), centrifuged at pH = 7.4 (13 000 g for 30 min at 4°C). Then, the supernatant comprising the ECPs was filtered successively through 0.45- and 0.22-mm pore-size Millipore Millex porosity filters and stored at −80°C until required. According to the manufacturer’s instructions, 5mg/ml bovine serum albumin (BSA) was used as the standard. Protein concentration of ECP was determined by Bradford protein assay (Kumar et al., 2019).

### Extracellular enzymatic activity assay

Using the agar plate punching method, sterile casein (0.4%), skimmed milk powder (0.4%), egg yolk (2.5%), soluble starch (0.2%), gelatin (0.4%), blood plate (containing 5% defibrillated sheep blood), urea (2.0%), and Tween-80 (1.0%) agar plate were prepared with ddH_2_O, respectively (Denkin and Nelson, 1999; Liu et al., 2012). The above materials were purchased from Lambolide Biotechnology Co., Ltd. At 28°C, the wild and knockout strains had the same activity of caseinase, protease, lecithinase, amylase, gelatinase, urease, and lipase. At the same time, the hemolytic activity of their extracellular products and the amount of protein were utterly consistent. A total of 10 μL sterile PBS (negative control) and the prepared extracellular products were added to the corresponding wells.

### Sequence alignment and homology analysis

Amino acid sequence alignment and homology analysis of *tatA, tatB*, and *tatC* from *A. salmonicida* SRW-OG1 were performed using NCBI database and biological software Clustalx 1.8. Then, A phylogenetic tree was constructed with neighbor-joining method using MEGA7.0 (XIAO et al., 2020).

### Prediction of protein secondary structure models

The virulence gene sequences were obtained from the *A. salmonicida* SRW-OG1 genome. With the I-TASSER, the protein secondary structure model was finally established and matched with all structures in the PDB library. A protein with the closest structural similarity was screened, which had the highest protein TM score (Yang and Zhang, 2015; Zhang et al., 2017).

### Statistical analysis

The expression quantitative software RSEM was used to analyze the gene expression level, calculate the correlation coefficient between each sample, and ensure the rationality of the experimental design. DESeq2 (http://bioconductor.org/packages/stats/bioc/DESeq2/) was used to detect the differential genes (DEGs) between the two samples, and use |log2FC|≥1 and q value<0.05 as the screening conditions. Statistical analysis was performed by one-way analysis of variance with Dunnett’s test using SPSS 22.0 software (Chicago, IL, USA). P < 0.05 was considered statistically significant.

## Results

### qRT-PCR validation of transcriptome data of *A. salmonicida* under different temperatures

**Figure 1:**
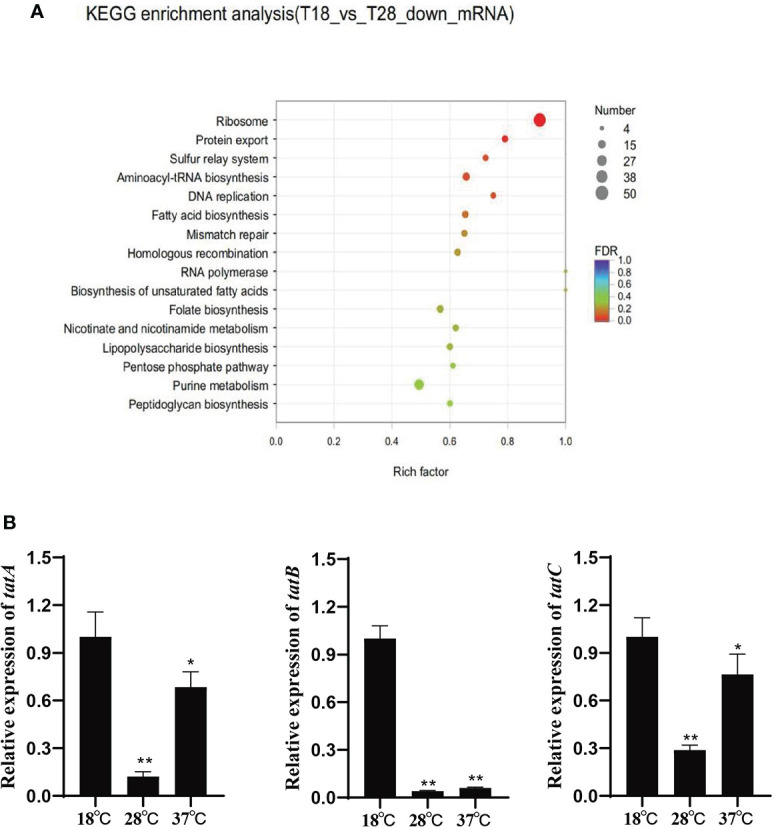
**(A)** Scatter plot of KEGG annotation distribution of differential genes; **(B)** Relative expression of *tatA*, *tatB* and *tatC* under different temperature stress, *P < 0.05, **P < 0.01.

Based on the KEGG pathway enrichment analysis of the differentially expressed genes under 18 and 28°C (**Figure 1A**), the down-regulated genes under 28°C were assigned to 16 different KEGG pathways, among which the protein export signaling pathway has been confirmed to be involved in the regulation of various virulence factors of pathogenic bacteria. In addition, the protein export signaling pathway is also a complex network regulation system. 15 differentially expressed genes enriched in this signaling pathway, including *tatA, tatB*, and *tatC*. They were significantly down-regulated under 28°C and slightly up-regulated under 37°C. The expression levels of *tatA, tatB* and *tatC* were verified by qRT-PCR (**Figure 1B**). The trend of gene expression level was consistent with the result of RNA-seq, indicating the reliability of RNA-seq.

### Amino acid sequence homology analysis of virulence genes

**Figure 2:**
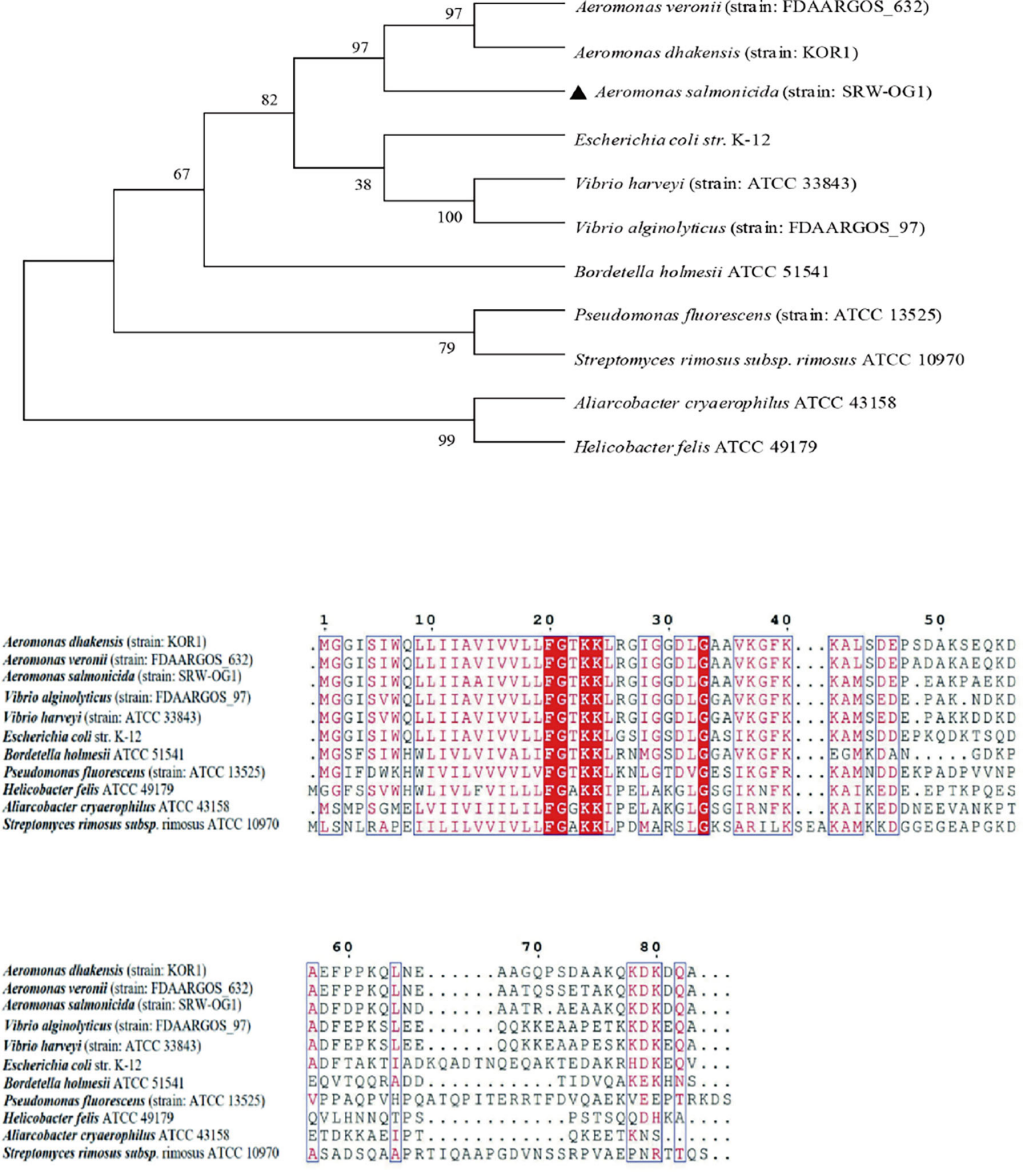
Phylogenetic tree of TatA amino acid sequence of type II secretion system.

To study the similarity of T2SS virulence genes among species, the amino acid sequences of TatA, TatB and TatC were analyzed. A total of 11 TatA sequences from *Aeromonas*, *Vibrio*, *Streptococcus* and *Pseudomonas* were selected to construct a phylogenetic tree by neighbor-joining method (N-J method). The results of multiple sequence alignment showed that the TatA in *A. salmonicida* SRW-OG1 was most similar to the *A. veronii* protein in the database, including the amino acid sequence of *A. dhakensis* from the same genus *Aeromonas* clustered into a branch; the amino acid sequences of TatA in *Aliarcobacter cryaerophilus* ATCC 4315 and *Helicobacter felis* ATCC 49179 are increasingly distant (**Figure 2**).

**Figure 3:**
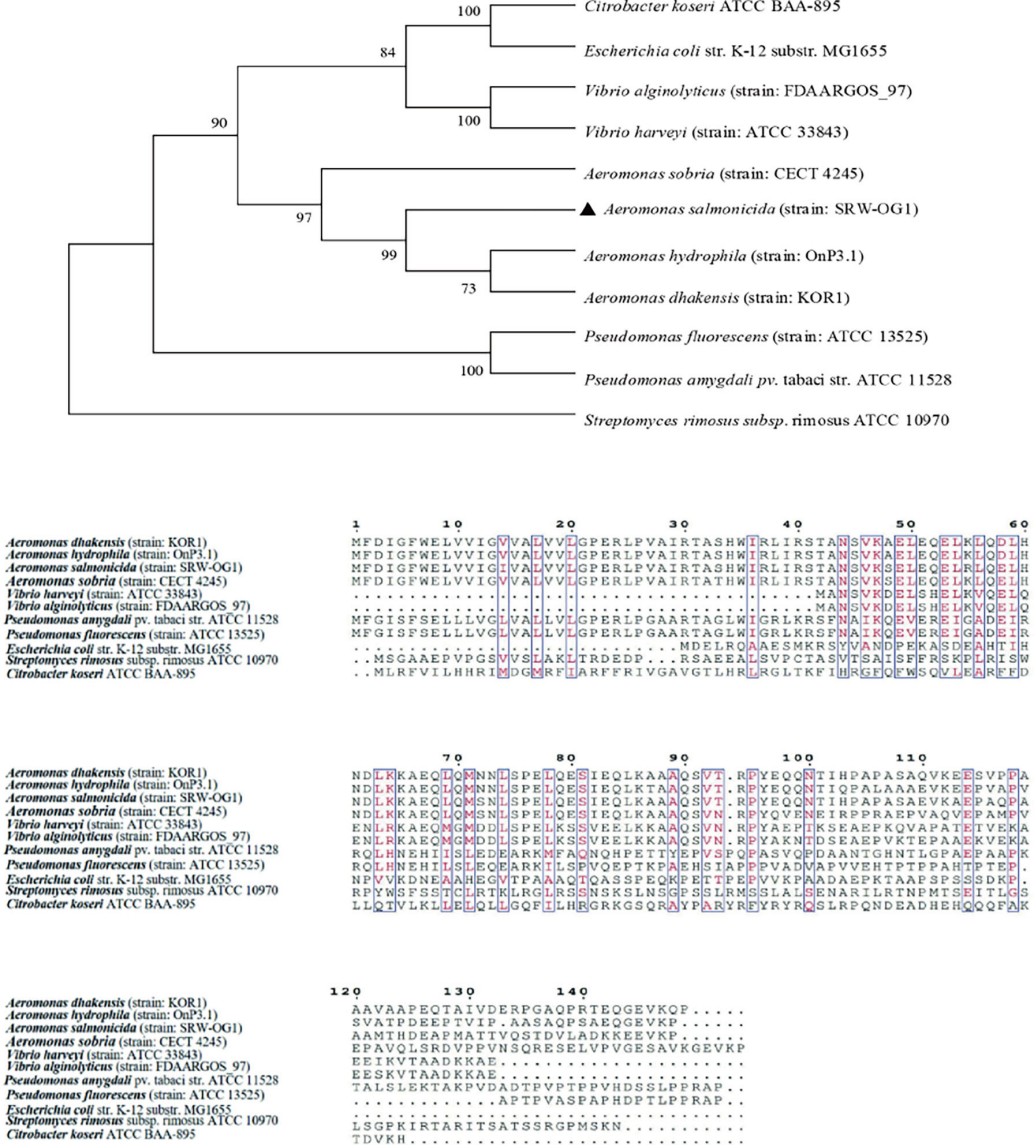
Phylogenetic tree of TatB amino acid sequence of type II secretion system.

The Neighbor-Joining method in Mega7.0 software was used for phylogenetic analysis of TatB amino acid sequences of the above different genera, and the default Poisson model was used. The results showed that the amino acid sequences in this study were clustered into a single branch, which had the closest genetic relationship with *Aeromonas hydrophila* (strain: OnP3.1) and *Aeromonas dhakensis* (strain: KOR1) with high conservation. *Streptomyces rimosus* subsp. rimosus ATCC 10970 and other sequences are obviously located in different branches (**Figure 3**).

**Figure 4:**
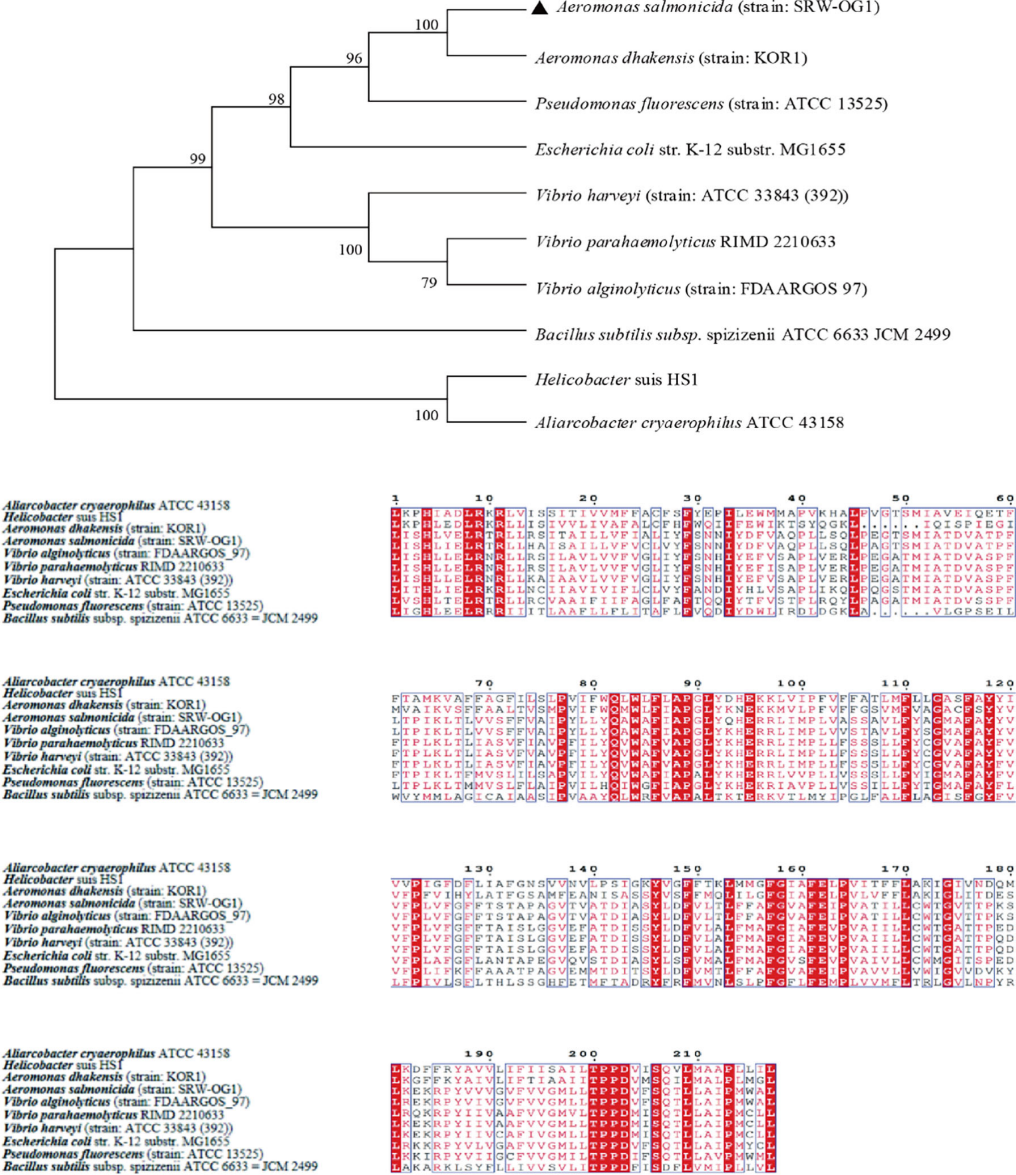
Phylogenetic tree of TatC amino acid sequence of type II secretion system.

10 TatC sequences from the genus *Monascus*, *E. coli*, and *Vibrio parahaemolyticus* were selected to construct an evolutionary tree. It can be seen from the phylogenetic tree: the amino acid sequence of TatC in this study and the sequence of *A. dhakensis* (strain: KOR1) belonging to the same family in the database were the most conserved and clustered together; while the *Helicobacter suis* HS1 sequence and the *Aliarcobacter cryaerophilus* ATCC 43158 sequence clustered into one branch and were far less conserved than the amino acid sequence from SRW-OG1;*Bacillus subtilis* subsp. spizizenii ATCC 6633 JCM 2499 was obviously located on a different branch from the sequences of *E. coli*, *Vibrio*, and *Salmonella* (**Figure 4**).

### Prediction of secondary structures of *TatA, TatB*, and *TatC*

**Figure 5:**
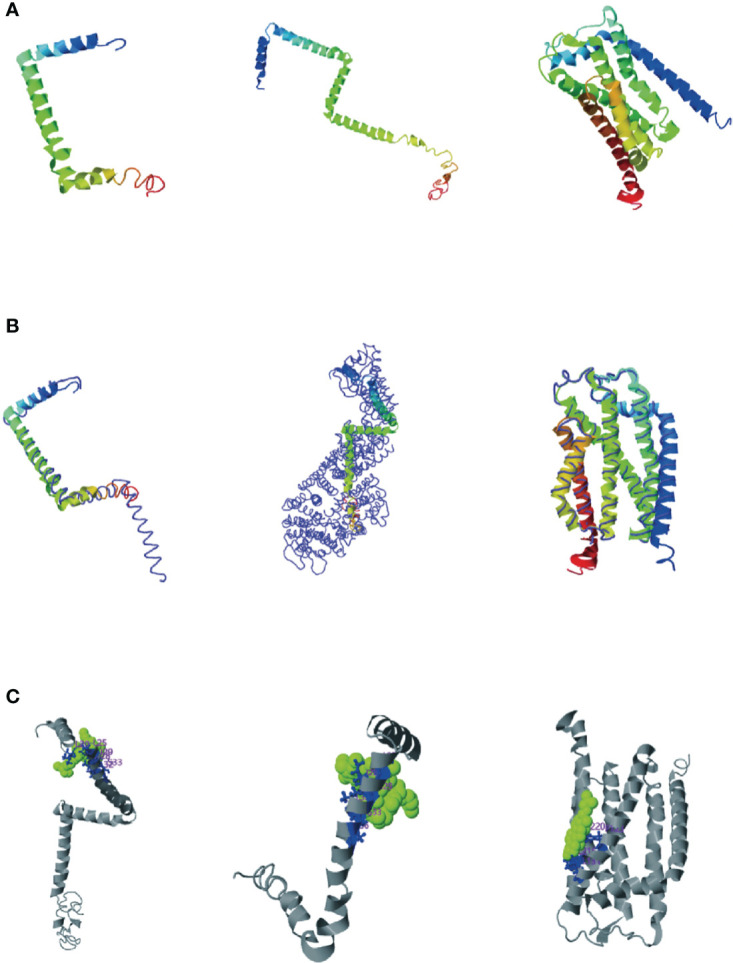
**(A)** Cluster protein structure model with the highest C-score value; **(B)** Proteins structurally close to the target in the PDB (as identified by TM-align); **(C)** COFACTOR and COACH analysis the ligand-binding site; The thin lines represent the backbone of the experimental structures, and the thick lines are the threading templates or the final models. Blue to red runs from N- to C-terminals.

Consensus-constrained and optimized I-TASSER used the SPICKER program to cluster all the decoys by pairwise structural similarity. They predicted protein structural models corresponding to the five most significant clusters. The confidence of each model was quantitatively measured by the C-score, which was calculated from the importance of thread template alignment and the convergence parameters of the structural assembly simulation (Li et al., 2022). The models were ranked from high C-score to low C-score, and the values were in the range [-5, 2]. The structural cluster protein structural model with the highest C-score value had the highest confidence, and vice versa. The highest C-scores of TatA, TatB, and TatC were -1.27, -1.27and 0.11, respectively (**Figure 5A**). The cluster protein structure model with the highest C-score value was matched with all structures in the PDB library, and the top 10 proteins whose structures were most similar were obtained, and they were arranged in descending order of TM-score. The protein with the highest TM score usually has a similar function to the target due to structural similarity, from which the biological function of the target gene can be predicted. The highest C-scores of TatA, TatB, and TatC were 0.673, 0.502and 0.865 (**Figure 5B**). While TM-align can derive functional annotations of the gene interested from global structural comparisons, analysis of ligand-binding sites using COFACTOR and COACH can better derive their biological functions from the multiplicity of sequence and structural features. The scores for the ligand-binding sites of TatA, TatB, and TatC were 0.13, 0.19and 0.09, respectively (**Figure 5C**).

The *tatA* of *A. salmonis* was most similar to the 2LZR protein Solution structure of the *E. coli* TatA protein in DPC micelles. 2LZRis an 89-residue monotopic integral membrane protein including a N-terminal transmembrane helix (TMH; corresponding to residue 5 - 20 in *E. coli* TatA), followed by a amphiphilic helix (APH; corresponding to residue 22 - 45 in *E. coli* TatA) and an unstructured and hydrophilic cytoplasm tail. TMH and APH form a right angle to each other, forming an “L” shape. The connection between the two helixes is centered on Gly21 (the “hinge brace”) (Rodriguez et al., 2013). The helix angle is the structural conservatism maintained by filling interaction (“hinge support”). The TatB of *A. salmonicida* were most similar in structure to the 2MI2 protein of *E. coli* (Solution structure of the *E. coli* TatB protein in DPC micelles). The structure of the 2MI2 protein is an extended “L-shape” consisting of four helical structures: a transmembrane helix (TMH) α1, an amphiphilic helix (APH) α2, and two solvent-exposed helices α3 and α4. The higher mobility of helices α3 and α4 makes them structurally conserved. TatC was most similar in structure to the 4B4A protein of *E. coli* (Structure of the TatC core of the twin arginine protein translocation system). The total structural weight of 4B4A protein is 29.43 kDa, which consists of 1873 atoms, its Length (Å) is a = 123.52, b = 123.52, c = 216.41, angle (°) α = 90, β = 90, γ = 120. The TatC exists as an integral membrane and does not allow significant ion leakage across the membrane, thus achieving the purpose of transporting only folded proteins to ensure the structural conservation of the Tat system.

### Enzymatic activity analysis of extracellular products under different culture temperatures

**Figure 6:**
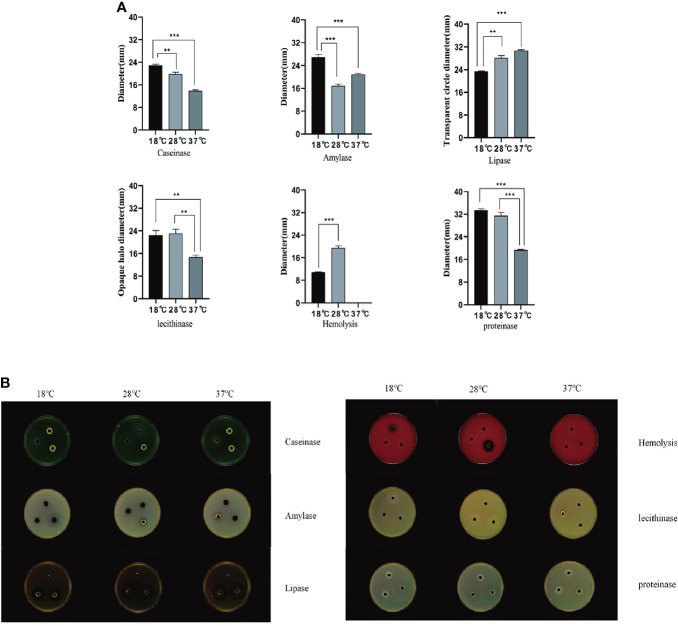
**(A)** Agar plate punching method to detect extracellular protease activity; **(B)** After the extracellular products were cultured on the agar plate for 24 hours, the b Shineso automatic colony counter was used for the transparent circle photographing test; The values marked by a express the mean of three independent experiments, and error bars represent standard deviation. Double and single asterisks indicate significant differences between wild strains at different temperatures (^**^ *P* < 0.05 and ^***^ *P* < 0.01), respectively.

In this study, three culture temperatures of 18°C, 28°C, and 37°C were selected to determine the enzymatic activity of the extracellular products of *A. salmonicida* SRW-OG1 (**Figure 6A**). The results showed that obvious activities of casease, amylase, lipase and lecithinase could be detected in the extracellular products of *A. salmonicida* SRW-OG1 under the three culture temperatures, but the activities of urease and gelatinase could not be detected. In addition, obvious transparent circles were observed at 18°C and 28°C, indicating that the extracellular products of *A. salmonicida* SRW-OG1 had hemolytic effect on sterile defibrillated sheep erythrocytes (**Table S4**).

It can be seen from (**Figure 6B**) that the ECP enzyme activity of *A. salmonicida* was significantly affected by temperature as follows: the activities of casein and protease at 18°C were significantly higher than those at 28°C (*P*< 0.05) and 37°C (*P*< 0.01). In addition, the amylase activity of *A. salmonicida* ECP at 18°C, 28°C, and 37°C was significantly different (*P*< 0. 05), which was the lowest at 28°C and the highest at 18°C. The lipase activity at 37°C was significantly higher than that at 18°C (P < 0.05); although the lipase activity measured at 28°C was slightly higher than that at 18°C, there was no significant difference between the two. The lecithinase activity measured at 37°C was significantly (*P*<0.05) lower than that at 18°C and 28°C; while at 18°C and 28°C, there was no significant difference between the two groups (*P*>0.05).

**Figure 7:**
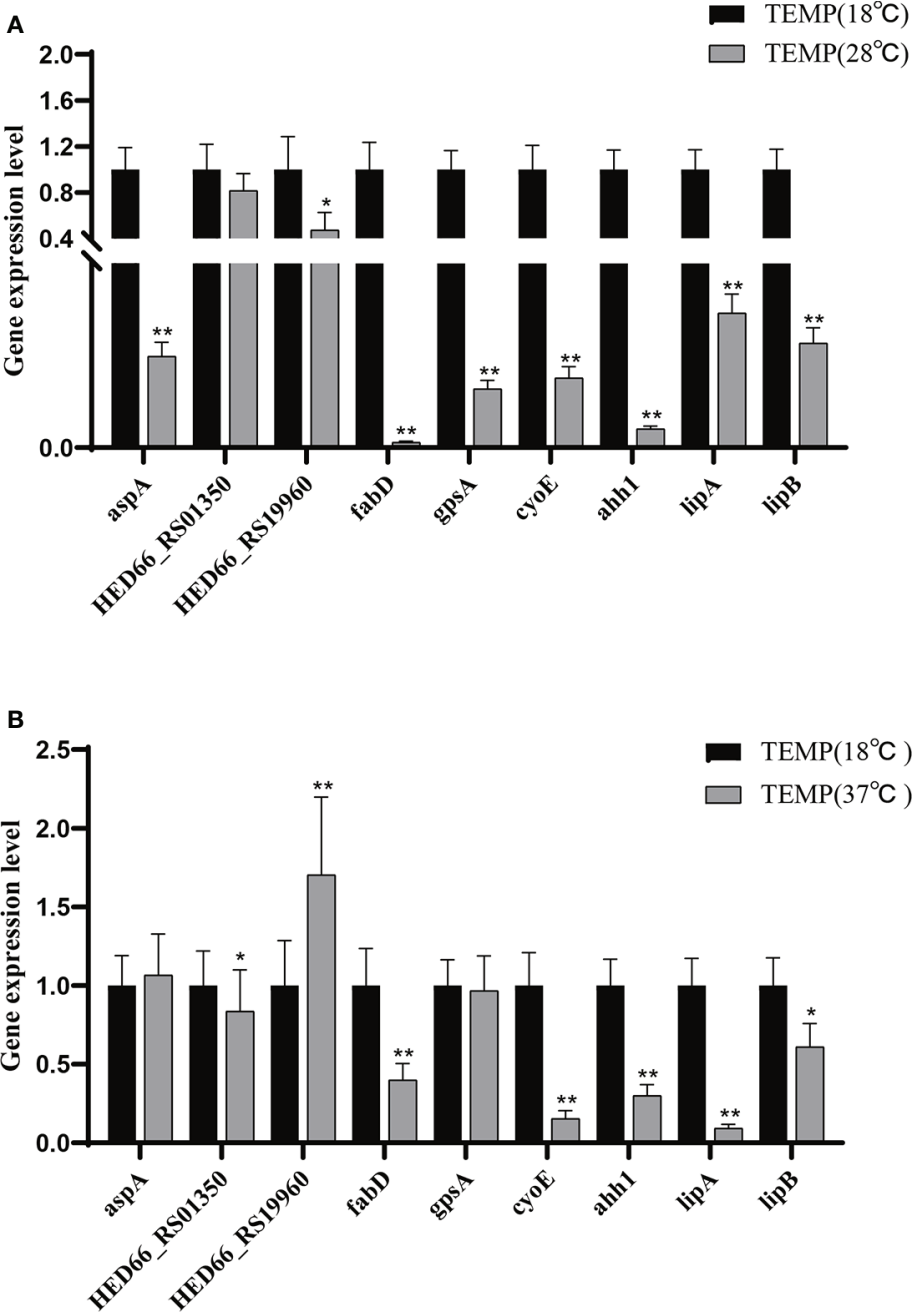
**(A)** Relative expression of extracellular product enzyme activity genes of *A. salmonicida* at 18°C - 28°C; **(B)** Relative expression of extracellular product enzyme activity genes of *A. salmonicida* at 18°C - 37°C, *P<0.05, **P<0.01.

The difference in extracellular enzyme activity may be caused by two reasons: (1) temperature affects the expression of genes related to the synthesis of extracellular enzymes; (2) temperature affects the secretion of extracellular enzymes. Analysis of the transcriptome of *A. salmonicida* under different temperatures showed that the expression levels of T2SS-related genes and some extracellular enzyme synthesis-related genes were significantly affected by temperature. To illustrate this, we detected the expression levels of genes regulating extracellular product-related enzyme activities by qRT-PCR. The experimental results showed that temperature stress had a significant effect on the expression of extracellular enzyme encoding genes in *A. salmonicida*. According to our results of enzyme activity analysis, we speculated that protease might be directly regulated by *aspA* (**Figure 7A**); *HED66 _ RS19960* played a major role in promoting the synthesis and secretion of amylase compared with *HED66 _ RS01350*; *fabD* and *gpsA* may be genes that directly synthesize lecithinase; hemolysis might be directly combined or co-regulated by *cyoE* and *ahh1*, so that *A. salmonicidal* cannot express hemolytic properties at 37°C; lipase might be promoted by *lipA* and *lipB*, resulting in low lipase secretion at 37°C (**Figure 7B**).

### Electrophoretic analysis of extracellular products

**Figure 8:**
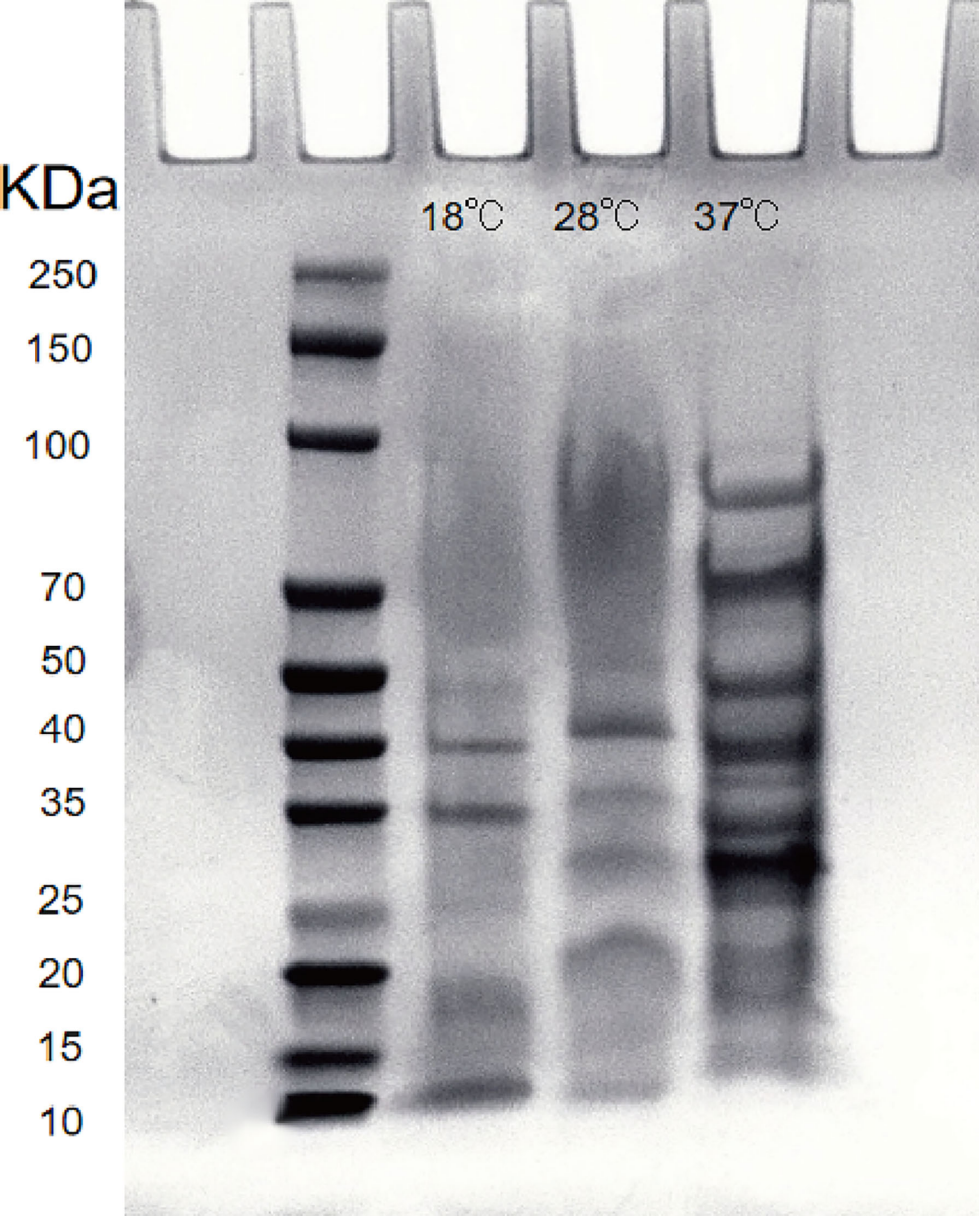
Lane 2: Protein marker; Lanel 3 - 5: Extracellular proteins of 18°Cˎ28°Cˎ3001;37°C, respectively; Lane 6: LB medium (negative control).

After the extracellular product (ECP) was extracted and confirmed to be free of bacteria, it was analyzed by SDS-PAGE electrophoresis. The Bradford protein concentration was determined by the known standard protein molecular mass (5mg/ml BSA) and its ECP, and the linear regression was performed to obtain the linear relationship equation (y=0.6995x+0.5958, R2 = 0.9912). The ECP protein concentration was adjusted to 1.2 mg/mL by PBS dilution. The results showed that the extracellular protein secretion of *A. salmonicida* was the lowest at 18°C, and the molecular weight of the product was 10-40 KDa. At 28°C, the extracellular protein secretion was more than that at 18°C, and the molecular weight of the product was 20-55 KDa. Extracellular protein secretion was the highest at 37°C, and its molecular weight was 15 ~ 90 KDa. The number and abundance of electrophoresis bands of *A. salmonicida* ECP extracted under different temperature stresses were quite different. However, the band with a molecular weight of 35KDa was a common band of ECP extracted at three different temperatures (**Figure 8**). Research about the specific differences through proteomics analysis was still necessary for future studies.

### Construction and identification of *tatA, tatB, tatC* mutants

**Figure 9:**
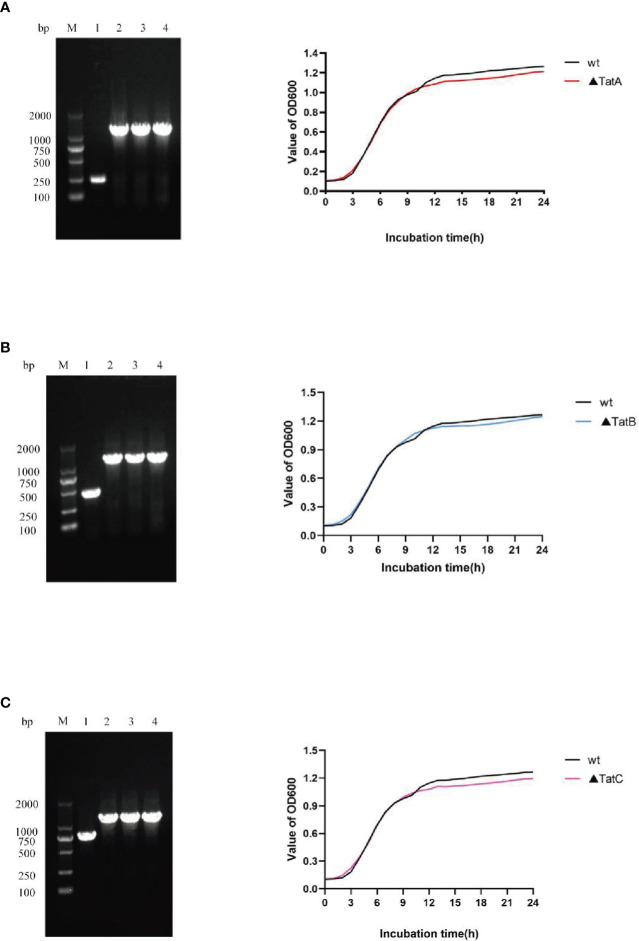
Construction and growth curve of Δ*tatA*, Δ*tatB*, and Δ*tatC* strains of *A. salmonicida,* Lane M: DNA molecular weight markers; Lanel 1: PCR products of wild strain; Lane 2 - 4: PCR products of *tatA* mutant **(A)**, PCR products of *tatB* mutant **(B)**, PCR products of *tatC* mutant **(C)**.

As described above, Δ*tatA*, Δ*tatB*, and Δ*tatC* were constructed. PCR amplification of SRW-OG1 was carried out with the identification primers on both sides of the target genes *tatA*, *tatB*, and *tatC*, and the sizes were 246 bp, 447 bp and 756 bp, respectively. PCR amplification of the respective gene in Δ*tatA*, Δ*tatB*, and Δ*tatC* obtained a fragment about 1200 bp. The growth curves of wild type and mutant strains were shown (**Figure 9**). Compared with that of wild type, the growth rate of mutants in the early stage was consistent with that of wild type, while the growth rate in the later stage was slightly lower than that of wild type, but there was no significant difference between the two.

### Effects of *tatA, tatB, and tatC* on virulence

**Figure 10:**
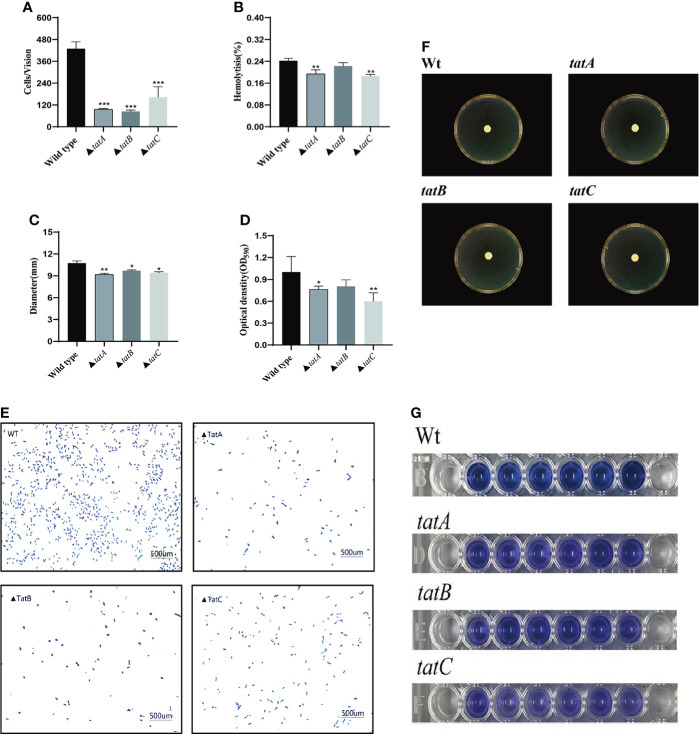
Characteristics of wild type, Δ*tatA*, Δ*tatB*, and Δ*tatC* mutants. Adhesion capacity **(A)**, hemolytic capacity **(B)**, motility **(C)**, and biofilm formation **(D)**, The effect on the adhesion ability of grouper mucus **(E)**, Motion phenotype **(F)**, biomembrane phenotype **(G)** were measured. Data are presented as mean ± SD. Three independent biological replicates were performed for each group. *P < 0.05, **P< 0.01.

**Figure 11:**
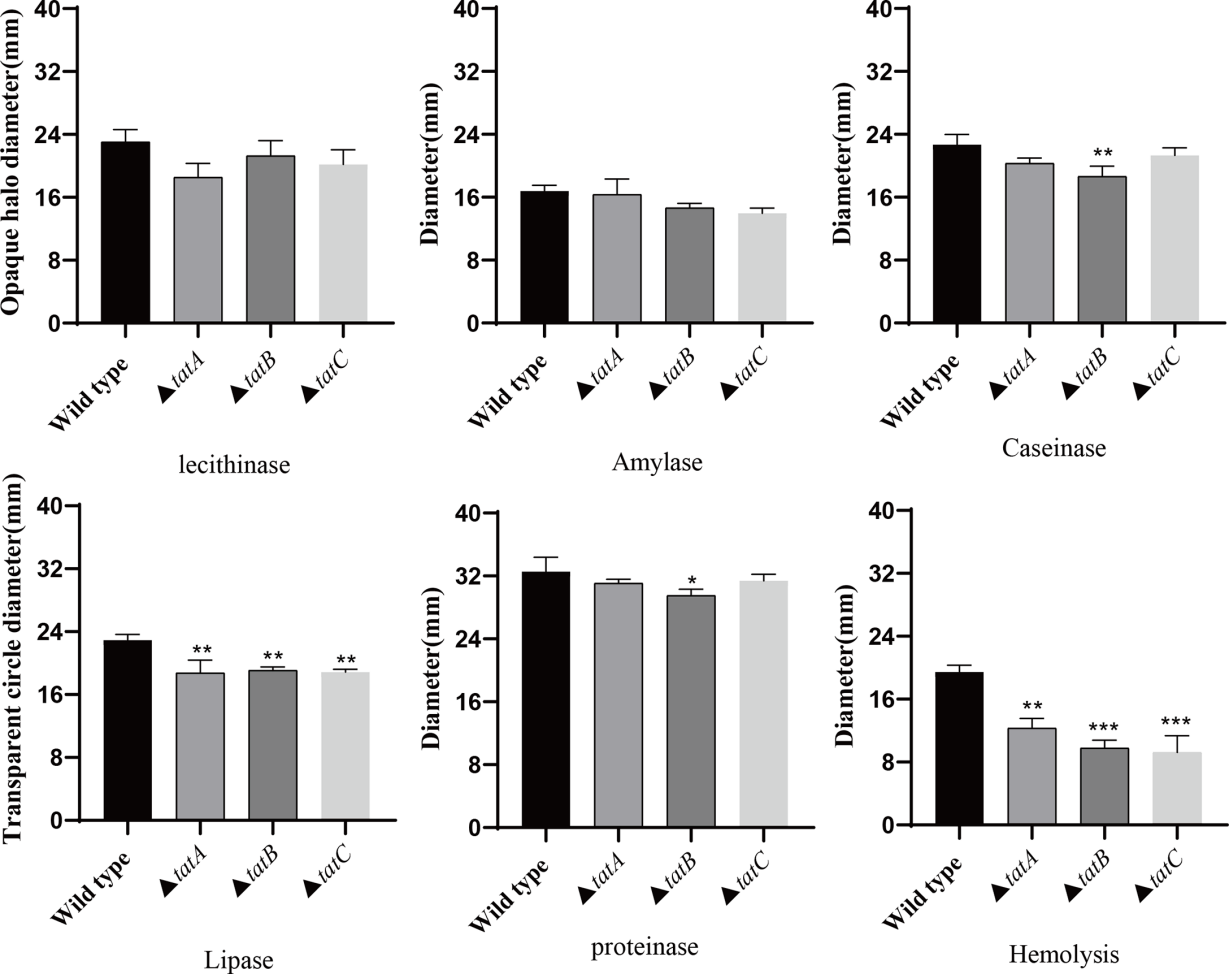
The extracellular enzyme activity characteristics of wild type, Δ*tatA*, Δ*tatB*, and Δ*tatC* mutants. Data are presented as mean ± SD. Three independent biological replicates were performed for each group. *P < 0.05, **P < 0.01, ***P< 0.001.

By comparing the adhesion (**Figure 10A**), hemolysis (**Figure 10B**), motility (**Figure 10C**), and biofilm formation (**Figure 10D**) of wild-type and *tatA, tatB*, and *tatC* mutant strains, the results showed that the number of adherent bacteria of the wild type, Δ*tatA*, Δ*tatB*, and Δ*tatC* strains were 429 ± 32, 95 ± 4, 84 ± 7 and 162 ± 48 cells/field (**Figure 10E**), we suggested that *tatA, tatB*, and *tatC* were involved in bacterial adhesion. The measurement results of the hemolytic ability showed that the hemolytic ability of the mutant strains decreased compared with the wild strain. In addition, when cultured on semi-solid agar for 12 hours, the colony diameter of *A. salmonicidal* mutant strains was markedly lower than that of wild strain. The average movement diameter of the wild type was 10.889 mm, the average movement diameter of the Δ*tatA* strain was 9.185 mm, the average movement diameter of the Δ*tatB* strain was 9.764 mm, and the average movement diameter of the Δ*tatC* strain was 9.490 mm, suggesting that these genes were associated with bacterial motility (**Figure 10F**). Compared with the wild-type strain, the mutant strains had insufficient bacterial biofilm formation ability during the entire biofilm formation process, especially the Δ*tatA* and Δ*tatC* showed significant reduction of biofilm formation (**Figure 10G**). Therefore, the *tatA, tatB*, and *tatC* genes had a significant positive effect on all four virulence phenotypes in *A. salmonicida.* In addition, the extracellular enzyme activities of wild type, Δ*tatA*, Δ*tatB*, and Δ*tatC* mutants were measured (**Figure 11**). The results showed that the extracellular products of wild type, Δ*tatA*, Δ*tatB*, and Δ*tatC* had obvious lecithinase, amylase, caseinase, lipase, protease and hemolysis activities, but the activities of urease and gelatinase could not be detected.

## Discussion

In our laboratory, *A. salmonicida* (SRW-OG1) was isolated from *Larimichthys Crocea* cultured at 28°C in Dongshan County, Zhangzhou City, Fujian Province. Most *A. salmonicida* are psychrophilic, but SRW-OG1 is mesophilic (Colquhoun and Sørum, 2001). As far as we know, temperature plays an important role in regulating secretion and activity of extracellular products of pathogenic bacteria, but some details remain unclear (Mateos et al., 1993; Khalil and Mansour, 1997; Ma et al., 2009). Studies on the extracellular products of pathogenic bacteria such as *Vibrio alginolyticus*, *Edwardsiella lentus*, *Aeromonas vermidis*, and *Aeromonas hydrophila* have discussed the influence of environmental factors on the enzymatic activity of extracellular products (Rojas et al., 2015).

There is increasing evidence that the extracellular protease of fish-derived *Vibrio alginolyticus* has an optimum temperature of 50°C, an optimum pH of 8.0, and poor thermal stability, indicating that the enzyme activity level of extracellular products secreted by pathogenic bacteria is affected by environmental factors, and the optimal reaction temperature is mainly in the range of 50-60°C (ZUO et al., 2006). Since the studies on extracellular products in these literatures are beyond the water temperature range of aquaculture, the stress temperature in this paper is set as pathogenic low temperature of 18°C, pathogenic high temperature of 28°C, and in virulent high temperature of 37°C. The results of this study showed that: (1) the activity of caseinase and protease at 18°C was significantly higher than that at 37°C; (2) The amylase activity was the lowest at 28°C and the highest at 18°C; (3) the lipase activity of ECP at 28°C and 37°C was significantly higher than that at 18°C; (4) the lipase activity measured at 28°C was slightly higher than that at 18°C, but there was no significant difference between the two; (5) the lecithinase activity measured at 37°C was significantly lower than that at 18°C and 28°C, but the difference in enzyme activity between the two at 18°C and 28°C was not significant. *A. salmonicida* ECP had a variety of enzyme activities, and most of the enzymes showed similar activities at 37°C, which was not pathogenic at high temperature, and 18°C, which was highly pathogenic at low temperature. Temperature affects the secretion of hemolytic enzyme through *cyoE* and *ahhh1*, thereby affecting the expression of extracellular hemolytic enzyme activity. *HED66_RS01350* and *HED66_RS19960* affect the synthesis of amylase, thereby affecting the expression of amylase activity. And *lipA* and *lipB* affect the synthesis of lipase, thereby affecting the expression of extracellular lipase activity. *FabD* and *gpsA* affect the secretion of lecithinase, thereby affecting the expression of lecithinase activity. It is revealed that the pathogenesis of boil disease in *Epinephelus coioides* is not limited to the expression of ECP enzyme activity, which provides a new idea for the treatment of *A. salmonicida*.

The Tat system were located in the protein secretion system of T2SS and function by secreting a fully folded protein that specifically recognizes a twin-arginine signal peptide (Wu et al., 2000). Meanwhile, the twin-arginine protein transport system (Tat), as a protein transport secretion system independent of the Sec system, is distributed on the inner membrane, and is closely related to many physiological functions of bacteria (Bogsch et al., 1998). Therefore, to further reveal the mechanism of Tat system affecting bacterial protein secretion, we constructed three mutants of Tat system. By analyzing the physiological phenotypes of *A. salmonicida tatA, tatB* and *tatC* mutant strains, combined with the results of bacterial virulence-related phenotypes and responses to temperature environmental stress, the intrinsic functional mechanisms of its transcriptional regulators were explored.

Studies once suggested that the Tat system has an essential effect on virulence in pathogenic bacteria such as *Salmonella* (Craig et al., 2013), *Yersinia pseudotuberculosis* (Avican et al., 2016), *Brucella melitensis* (Yan et al., 2020) and so on. In *Burkholderia thailandensis*, research has shown that the Tat system is vital for aerobic but not anaerobic growth (Wagley et al., 2014). However, in this study, the gene knockout of *tatA, tatB* and *tatC* did not affect the virulence of *A. salmonicida* by affecting the growth ability. The growth tolerance of mutant strains was consistent with the growing trend of wild strains. Furthermore, this study proved that after the deletion of the *tatA, tatB* and *tatC* genes, the number of the mutant strains in the mucus of the grouper was significantly lower than that of the wild strain. The relative reduction of *tatA* was 77.69%, *tatB* was 80.41%and *tatC* was 62.14%. These results indicated that the *tatA, tatB* and *tatC* genes in the type II secretion system of *A. salmonicida* played important roles in the regulatory network in response to changes in environmental factors under different environmental conditions (Cléon et al., 2015).

In addition, in *Dickeya zeae*, *otatA, otatB* and *otatE* mutants significantly reduced motility and failed to form biofilms, while the *otatC* mutant did not show a significant reduction in motility and biofilm formation (Zhang et al., 2018). We determined the swarming motility, biofilm formation, and hemolytic capacity of the mutant strains. The results showed no significant change in the motility compared with the wild type; however, the biofilm formation ability was weakened, which indicated that *tatA, tatB*, and *tatC* were involved in the biofilm formation process of *A. salmonicida*. Meanwhile, when we compared the difference in hemolytic ability between the wild-type and mutant strains, we found that the hemolytic activity of the *tatC* mutant strain was the most reduced by 23.41%. Several genes regulate the expression of virulence factor-related proteins and lead to changes in bacterial hemolysis, thereby participating in the regulation of bacterial virulence (Armbruster et al., 2019).

In conclusion, this study reported for the first time the expression mode of *tatA, tatB*, and *tatC* genes in T2SS of *A. salmonicida* under different temperatures. It preliminarily confirmed their essential roles in virulence regulation. The genes affecting *A. salmonicida* amylase, protease, lipase, hemolytic ability, and lecithinase were also identified as *cyoE, ahh1, lipA, lipB, pulA*, *HED66_RS01350, HED66_RS19960, aspA, fabD*, and *gpsA*. The results of this study could provide a new theoretical reference for the study of the pathogenesis of *A. salmonicida* and the formulation of prevention and treatment strategies.

## Data availability statement

The original contributions presented in the study are included in the article/supplementary material. Further inquiries can be directed to the corresponding authors.

## Ethics statement

All laboratory animals were operated on according to the guidelines in the “Guidelines for the Care and Use of Laboratory Animals” developed by the National Institutes of Health. The animal experiments were approved by Jimei University Animal Ethics Committee (Acceptance NO: JMULAC201159).

## Author contributions

XY and YC are responsible for sequencing and article writing. HC, JW, and ZZ are responsible for the collection and processing 17 text pages. XX, YZ, ML, YQ, and XJ are responsible for the experimental design. All authors contributed to the article and approved the submitted version.

## Funding

This research was supported by the Natural Science Foundation of Fujian Province (Project No. 2020J01673, 2019J01695), [2020] No.32, ZP2021001, The Scientific Research Fund of Engineering Research Center of the Modern Industry Technology for Eel Ministry of Education (Project No. RE202108), Xiamen Ocean and Fishery Development Special Fund (21CZP007HJ07), Open Research Fund Project of State Key Laboratory of Large Yellow Croaker Breeding (Project No. LYC2018RS04), the National Key Research and Development Program of China (Project No. 2018YFC1406305), the Foreign Cooperation Project of Fujian Province (Project No. 2019I1008), the Science and Technology Platform Construction of Fujian Province (Project No. 2018N2005, 2017L3019), the NSFC (General Program Project No. 31702384), the Scientific Research Fund of Fujian Provincial Department of Education (Project No. JA15292), and the open fund of the Fujian Province Key Laboratory of Special Aquatic Formula Feed (Fujian Tianma Science and Technology Group Co., Ltd. Project No. TMKJZ1907), Science and Technology Commissioner of Fujian Province (Project No. MinKeNong [2019] No.11, ZP2021001), and The National Key Research and Development Plan (Project No. 2020YFD0900102).

## Conflict of interest

The authors declare that the research was conducted in the absence of any commercial or financial relationships that could be construed as a potential conflict of interest.

## Publisher’s note

All claims expressed in this article are solely those of the authors and do not necessarily represent those of their affiliated organizations, or those of the publisher, the editors and the reviewers. Any product that may be evaluated in this article, or claim that may be made by its manufacturer, is not guaranteed or endorsed by the publisher.

## References

[B1] Armbruster C. E. Forsyth V. S. Johnson A. O. Smith S. N. White A. N. Brauer A. L.. (2019). Twin arginine translocation, ammonia incorporation, and polyamine biosynthesis are crucial for Proteus mirabilis fitness during bloodstream infection. PloS Pathog. 15 (4), e1007653. doi: 10.1371/journal.ppat.1007653 31009518 PMC6497324

[B2] Austin B. Austin D. A. Munn C. (2007). Bacterial fish pathogens: disease of farmed and wild fish (Springer).

[B3] Austin B. Rodgers C. (1980). Preliminary observations on aeromonas salmonicida vaccines. Developments Biol. standardization.

[B4] Avican U. Beckstette M. Heroven A. K. Lavander M. Dersch P. Forsberg Å. (2016). Transcriptomic and phenotypic analysis reveals new functions for the tat pathway in yersinia pseudotuberculosis. J. bacteriol. 198 (20), 2876–2886. doi: 10.1128/JB.00352-16 27501981 PMC5038016

[B5] Bhat R. A. H. Thakuria D. Dubey M. K. Tandel R. S. Sharma P. Khangembam V. C.. (2021). Lethal dose and histopathological alterations induced by aeromonas salmonicida in experimentally challenged common carp, cyprinus carpio. Microbial Pathogenesis 158, 105110. doi: 10.1016/j.micpath.2021.105110 34314809

[B6] Bogsch E. G. Sargent F. Stanley N. R. Berks B. C. Robinson C. Palmer T. (1998). An essential component of a novel bacterial protein export system with homologues in plastids and mitochondria. J. Biol. Chem. 273 (29), 18003–18006. doi: 10.1074/jbc.273.29.18003 9660752

[B7] Buck E. D. Maes L. Robben J. Noben J. P. Anné J. Lammertyn E. (2006). Identification of putative substrates of the legionella pneumophila tat secretion pathway via two-dimensional protein gel electrophoresis. Legionella: State Art 30 Years after Its Recognition 217–220. doi: 10.1128/9781555815660.ch54

[B8] Cléon F. Habersetzer J. Alcock F. Kneuper H. Stansfeld P. J. Basit H.. (2015). The TatC component of the twin-arginine protein translocase functions as an obligate oligomer. Mol. Microbiol. 98 (1), 111–129. doi: 10.1111/mmi.13106 26112072 PMC5102672

[B9] Colquhoun D. Sørum H. (2001). Temperature dependent siderophore production in vibrio salmonicida. Microbial pathogenesis 31 (5), 213–219. doi: 10.1006/mpat.2001.0464 11710841

[B10] Craig M. Sadik A. Y. Golubeva Y. A. Tidhar A. Slauch J. M. (2013). Twin-arginine translocation system (tat) mutants of salmonella are attenuated due to envelope defects, not respiratory defects. Mol. Microbiol. 89 (5), 887–902. doi: 10.1111/mmi.12318 23822642 PMC3811912

[B11] Datsenko K. A. Wanner B. L. (2000). One-step inactivation of chromosomal genes in escherichia coli K-12 using PCR products. Proc. Natl. Acad. Sci. 97 (12), 6640–6645. doi: 10.1073/pnas.120163297 10829079 PMC18686

[B12] Denkin S. M. Nelson D. R. (1999). Induction of protease activity in vibrio anguillarum by gastrointestinal mucus. Appl. Environ. Microbiol. 65 (8), 3555–3560. doi: 10.1128/AEM.65.8.3555-3560.1999 10427048 PMC91533

[B13] Ding Z. Christie P. J. (2003). Agrobacterium tumefaciens twin-Arginine-Dependent translocation is important for virulence, flagellation, and chemotaxis but not type IV secretion. J. bacteriol. 185 (3), 760–771. doi: 10.1128/JB.185.3.760-771.2003 12533451 PMC142831

[B14] Du Y. Yi M. Xiao P. Meng L. Li X. Sun G.. (2015). The impact of aeromonas salmonicida infection on innate immune parameters of Atlantic salmon (Salmo salar l). Fish shellfish Immunol. 44 (1), 307–315. doi: 10.1016/j.fsi.2015.02.029 25725402

[B15] Fernandez L. Lopez J. Secades P. Menendez A. Marquez I. Guijarro J. (2003). *In vitro* and *in vivo* studies of the Yrp1 protease from yersinia ruckeri and its role in protective immunity against enteric red mouth disease of salmonids. Appl. Environ. Microbiol. 69 (12), 7328–7335. doi: 10.1128/AEM.69.12.7328-7335.2003 14660382 PMC309943

[B16] González E. T. Brown D. G. Swanson J. K. Allen C. (2007). Using the ralstonia solanacearum tat secretome to identify bacterial wilt virulence factors. Appl. Environ. Microbiol. 73 (12), 3779–3786. doi: 10.1128/AEM.02999-06 17468289 PMC1932711

[B17] Guijarro J. A. Cascales D. García-Torrico A. I. García-Domínguez M. Méndez J. (2015). Temperature-dependent expression of virulence genes in fish-pathogenic bacteria. Front. Microbiol. 6, 700. doi: 10.3389/fmicb.2015.00700 26217329 PMC4496569

[B18] He R. Wang J. Lin M. Tian J. Wu B. Tan X.. (2022). Effect of ferredoxin receptor FusA on the virulence mechanism of pseudomonas plecoglossicida. Front. Cell. infection Microbiol. 255. doi: 10.3389/fcimb.2022.808800 PMC8981516 35392610

[B19] He R. Zhao L. Xu X. Zheng W. Zhang J. Zhang J.. (2020). Aryl hydrocarbon receptor is required for immune response in epinephelus coioides and danio rerio infected by pseudomonas plecoglossicida. Fish Shellfish Immunol. 97, 564–570. doi: 10.1016/j.fsi.2019.12.084 31891808

[B20] Holden N. J. Uhlin B. E. Gally D. L. (2001). PapB paralogues and their effect on the phase variation of type 1 fimbriae in escherichia coli. Mol. Microbiol. 42 (2), 319–330. doi: 10.1046/j.1365-2958.2001.02656.x 11703657

[B21] Huang L. Qiao Y. Xu W. Gong L. He R. Qi W.. (2021a). Full-length transcriptome: A reliable alternative for single-cell RNA-seq analysis in the spleen of teleost without reference genome. Front. Immunol. 3974. doi: 10.3389/fimmu.2021.737332 PMC8502891 34646272

[B22] Huang L. Qi W. Zuo Y. Alias S. A. Xu W. (2020a). The immune response of a warm water fish orange-spotted grouper (Epinephelus coioides) infected with a typical cold water bacterial pathogen aeromonas salmonicida is AhR dependent. Dev. Comp. Immunol. 113, 103779. doi: 10.1016/j.dci.2020.103779 32735958

[B23] Huang L. Zhao L. Qi W. Xu X. Zhang J. Yan Q. (2020). Temperature-specific expression of cspA1 contributes to activation of sigX during pathogenesis and intracellular survival in Pseudomonas plecoglossicida. Aquaculture 518, 734861.

[B24] Huntingford F. Adams C. Braithwaite V. Kadri S. Pottinger T. Sandøe P.. (2007). Erratum: Current issues in fish welfare (Journal of fish biology (2006) 68 (332-372). J. Fish Biol. 70 (4), 1311–1316. doi: 10.1111/j.0022-1112.2006.001046.x

[B25] Khalil A. Mansour E. (1997). Toxicity of crude extracellular products of aeromonas hydrophila in tilapia, tilapianilotica. Lett. Appl. Microbiol. 25 (4), 269–273. doi: 10.1046/j.1472-765X.1997.00220.x 9351277

[B26] Kobayashi T. Goto K. Miyazaki T. (2000). Pathological changes caused by cold-water stress in Japanese eel Anguilla japonica. Dis. Aquat. organisms 40 (1), 41–50. doi: 10.3354/dao040041 10785862

[B27] Kumar V. Nguyen D. V. Baruah K. Bossier P. (2019). Probing the mechanism of VP_AHPND_ extracellular proteins toxicity purified from vibrio parahaemolyticus AHPND strain in germ-free artemia test system. Aquaculture 504, 414–419. doi: 10.1016/j.aquaculture.2019.02.029

[B28] Li G. An T. Li Y. Yue J. Huang R. Huang J.. (2022). Transcriptome analysis and identification of the cholesterol side chain cleavage enzyme BbgCYP11A1 from bufo bufo gargarizans. Front. Genet. 13, 828877. doi: 10.3389/fgene.2022.828877 35480310 PMC9037069

[B29] Li H. Qin Y. Mao X. Zheng W. Luo G. Xu X.. (2019). Silencing of cyt-c4 led to decrease of biofilm formation in aeromonas hydrophila. Bioscience Biotechnol. Biochem. 83 (2), 221–232. doi: 10.1080/09168451.2018.1528543 30304991

[B30] Liu H. Gu D. Cao X. Liu Q. Wang Q. Zhang Y. (2012). Characterization of a new quorum sensing regulator luxT and its roles in the extracellular protease production, motility, and virulence in fish pathogen vibrio alginolyticus. Arch. Microbiol. 194 (6), 439–452. doi: 10.1007/s00203-011-0774-x 22130678

[B31] Ma L. Chen J. Liu R. Zhang X.-H. Jiang Y.-A. (2009). Mutation of rpoS gene decreased resistance to environmental stresses, synthesis of extracellular products and virulence of vibrio anguillarum. FEMS Microbiol. Ecol. 70 (2), 286–292. doi: 10.1111/j.1574-6941.2009.00713.x 19527291

[B32] Mateos D. Anguita J. Naharro G. Paniagua C. (1993). Influence of growth temperature on the production of extracellular virulence factors and pathogenicity of environmental and human strains of aeromonas hydrophila. J. Appl. Bacteriol. 74 (2), 111–118. doi: 10.1111/j.1365-2672.1993.tb03003.x 8444639

[B33] Maurice S. Tinman S. (2000). First observations of carp erythrodermatitis caused by atypical aeromonas salmonicida in Israeli bred cyprinus carpio. Israeli J. Aquaculture/Bamidgeh 52 (1), 36–45.

[B34] Méndez J. Guijarro J. (2013). *In vivo* monitoring of yersinia ruckeri in fish tissues: progression and virulence gene expression. Environ. Microbiol. Rep. 5 (1), 179–185. doi: 10.1111/1758-2229.12030 23757147

[B35] Meng L. Du Y. Liu P. Li X. Liu Y. (2017). Involvement of LuxS in aeromonas salmonicida metabolism, virulence and infection in Atlantic salmon (Salmo salar l). Fish Shellfish Immunol. 64, 260–269. doi: 10.1016/j.fsi.2017.03.009 28279794

[B36] Merino S. Tomás J. M. (2016). The aeromonas salmonicida lipopolysaccharide core from different subspecies: The unusual subsp. pectinolytica. Front. Microbiol. 7, 125. doi: 10.3389/fmicb.2016.00125 26904002 PMC4749718

[B37] Murphy K. C. (1998). Use of bacteriophage lambda recombination functions to promote gene replacement in escherichia coli. J. Bacteriol. 180 (8), 2063–2071. doi: 10.1128/JB.180.8.2063-2071.1998 9555887 PMC107131

[B38] Ochsner U. A. Snyder A. Vasil A. I. Vasil M. L. (2002). Effects of the twin-arginine translocase on secretion of virulence factors, stress response, and pathogenesis. Proc. Natl. Acad. Sci. 99 (12), 8312–8317. doi: 10.1073/pnas.082238299 12034867 PMC123064

[B39] Palmer T. Berks B. C. (2012). The twin-arginine translocation (Tat) protein export pathway. Nat. Rev. Microbiol. 10 (7), 483–496. doi: 10.1038/nrmicro2814 22683878

[B40] Qi W. Gao Q. Tian J. Wu B. Lin M. Qi S.. (2022). Immune responses and inorganic ion transport regulations of epinephelus coioides in response to L321_RS13075 gene of pseudomonas plecoglossicida. Fish Shellfish Immunol. 120, 599–609. doi: 10.1016/j.fsi.2021.12.036 34968707

[B41] Rodriguez F. Rouse S. L. Tait C. E. Harmer J. De Riso A. Timmel C. R.. (2013). Structural model for the protein-translocating element of the twin-arginine transport system. Proc. Natl. Acad. Sci. 110 (12), E1092–E1101. doi: 10.1073/pnas.1219486110 23471988 PMC3607022

[B42] Rojas R. Miranda C. D. Opazo R. Romero J. (2015). Characterization and pathogenicity of vibrio splendidus strains associated with massive mortalities of commercial hatchery-reared larvae of scallop argopecten purpuratus (Lamarck, 1819). J. Invertebrate Pathol. 124, 61–69. doi: 10.1016/j.jip.2014.10.009 25450196

[B43] Rupp M. Pilo P. Müller B. Knüsel R. von Siebenthal B. Frey J.. (2019). Systemic infection in European perch with thermoadapted virulent aeromonas salmonicida (Perca fluviatilis). J. fish Dis. 42 (5), 685–691. doi: 10.1111/jfd.12970 30806486

[B44] Salomón R. Furones M. D. Reyes-López F. E. Tort L. Firmino J. P. Esteban M. A.. (2021). A bioactive extract rich in triterpenic acid and polyphenols from olea europaea promotes systemic immunity and protects Atlantic salmon smolts against furunculosis. Front. Immunol. 12. doi: 10.3389/fimmu.2021.737601 PMC8633542 34867959

[B45] Sformo T. L. de la Bastide P. Y. LeBlanc J. Givens G. H. Adams B. Seigle J. C.. (2021). Temperature response and salt tolerance of the opportunistic pathogen saprolegnia parasitica: Implications for the broad whitefish subsistence fishery. Arctic Antarctic Alpine Res. 53 (1), 271–285. doi: 10.1080/15230430.2021.1970340

[B46] Soto-Dávila M. Hossain A. Chakraborty S. Rise M. L. Santander J. (2019). Aeromonas salmonicida subsp. salmonicida early infection and immune response of Atlantic cod (Gadus morhua l.) primary macrophages. Front. Immunol. 1237. doi: 10.3389/fimmu.2019.01237 PMC6559310 31231379

[B47] Stanley N. R. Findlay K. Berks B. C. Palmer T. (2001). Escherichia coli strains blocked in tat-dependent protein export exhibit pleiotropic defects in the cell envelope. J. Bacteriol. 183 (1), 139–144. doi: 10.1128/JB.183.1.139-144.2001 11114910 PMC94859

[B48] Vasquez I. Cao T. Hossain A. Valderrama K. Gnanagobal H. Dang M.. (2020). Aeromonas salmonicida infection kinetics and protective immune response to vaccination in sablefish (Anoplopoma fimbria). Fish Shellfish Immunol. 104, 557–566. doi: 10.1016/j.fsi.2020.06.005 32592927

[B49] Voulhoux R. Ball G. Ize B. Vasil M. L. Lazdunski A. Wu L.-F.. (2001). Involvement of the twin-arginine translocation system in protein secretion via the type II pathway. EMBO J. 20 (23), 6735–6741. doi: 10.1093/emboj/20.23.6735 11726509 PMC125745

[B50] Wagley S. Hemsley C. Thomas R. Moule M. G. Vanaporn M. Andreae C.. (2014). The twin arginine translocation system is essential for aerobic growth and full virulence of burkholderia thailandensis. J. Bacteriol. 196 (2), 407–416. doi: 10.1128/JB.01046-13 24214943 PMC3911251

[B51] Wu L.-F. Ize B. Chanal A. Quentin Y. Fichant G. (2000). Bacterial twin-arginine signal peptide-dependent protein translocation pathway: Evolution and mechanism. J. Mol. Microbiol. Biotechnol. 2 (2), 179–189. doi: 10.1038/sj.jim.2900821 10939242

[B52] Wu J. Kim K.-S. Lee J.-H. Lee Y.-C. (2010). Cloning, expression in escherichia coli, and enzymatic properties of laccase from aeromonas hydrophila WL-11. J. Environ. Sci. 22 (4), 635–640. doi: 10.1016/S1001-0742(09)60156-X 20617743

[B53] XIAO W. WANG X. JIANG Y. SUN M. CHANG Y. QU Y.. (2020). Characteristics of plasmids in KPC-2-producing serratia marcescens. Chin. J. Microbiol. Immunol., 757–762. doi: 10.3760/cma.j.cn112309-20200309-00107

[B54] Xu Z. Jin P. Zhou X. Zhang Y. Wang Q. Liu X.. (2021). Isolation of a virulent aeromonas salmonicida subsp. masoucida bacteriophage and its application in phage therapy in turbot (Scophthalmus maximus). Appl. Environ. Microbiol. 87 (21), e01468–e01421. doi: 10.1128/AEM.01468-21 34406829 PMC8516056

[B55] Xu K.-Z. Tan X.-J. Chang Z.-Y. Li J.-J. Jia A.-Q. (2022). 2-tert-Butyl-1, 4-benzoquinone, a food additive oxidant, reduces virulence factors of chromobacterium violaceum. LWT 113569. doi: 10.1016/j.lwt.2022.113569

[B56] Yang J. Zhang Y. (2015). I-TASSER server: new development for protein structure and function predictions. Nucleic Acids Res. 43 (W1), W174–W181. doi: 10.1128/IAI.00389-20 25883148 PMC4489253

[B57] Yan X. Hu S. Yang Y. Xu D. Li H. Liu W.. (2020). The twin-arginine translocation system is important for stress resistance and virulence of brucella melitensis. Infection Immun. 88 (11), e00389–e00320. doi: 10.1128/IAI.00389-20 PMC7573438 32778612

[B58] Zhang X. H. Austin B. (2000). Pathogenicity of vibrio harveyi to salmonids. J. fish Dis. 23 (2), 93–102. doi: 10.1046/j.1365-2761.2000.00214.x

[B59] Zhang C. Freddolino P. L. Zhang Y. (2017). COFACTOR: improved protein function prediction by combining structure, sequence and protein–protein interaction information. Nucleic Acids Res. 45 (W1), W291–W299. doi: 10.1093/nar/gkx366 28472402 PMC5793808

[B60] Zhang Q. Yu C. Wen L. Liu Q. (2018). Tat system is required for the virulence of dickeya zeae on rice plants. J. Plant Pathol. 100 (3), 409–418. doi: 10.1007/s42161-018-0086-y

[B61] Zhong Y. Qi W. Xu W. Zhao L. Xiao B. Yan Q.. (2021). Insights into mesophilic virulence, antibiotic resistant and human pathogenicity: A genomics study on the aeromonas salmonicida SRW-OG_1_ newly isolated from the Asian fish epinephelus coioides. Aquaculture 539, 736630. doi: 10.1016/j.aquaculture.2021.736630

[B62] ZUO F.-Q. JIAN J.-C. WU Z.-H. (2006). Characterization of extracellular products from vibrio alginolyticus isolated from maricultured fish. Acta Hydrobiol. Sin. 05), 553–558. doi: 10.1016/S0379-4172(06)60053-X

[B63] Zuo Y. Zhao L. Xu X. Zhang J. Zhang J. Yan Q.. (2019). Mechanisms underlying the virulence regulation of new vibrio alginolyticus ncRNA Vvrr1 with a comparative proteomic analysis. Emerging Microbes infections 8 (1), 1604–1618. doi: 10.1080/22221751.2019.1687261 31711375 PMC6853220

