# Supplementary material for: The temperature-dependent expression of type II secretion system controls extracellular product secretion and virulence in mesophilic Aeromonas salmonida SRW-OG1
Source: Front Cell Infect Microbiol. 2022 Aug 1;12:945000. doi: 10.3389/fcimb.2022.945000 (PMC9376225; doi:10.3389/fcimb.2022.945000)
Supplement: Supplementary file 1 [file Table_1.docx]

Table S1. Targeted fragments and primer sequences for knockouts

| Target gene | Primers sequence for gene overexpression |
| --- | --- |
| *tatA*-mut-F | 5’-ATGGGTGGTAGTGAAACCCAACATACCCC-3’ |
| *tatA*-mut-R | 5’-TTAAGCCTGATCAGCGATCGGCTCG-3’ |
| *tatB*-mut-F | 5’-ATGTTCGATAGTGAAACCCAACATACCCC-3’ |
| *tatB*-mut-R | 5’- TCATGGCTTCTCAGCGATCGGCTCG -3’ |
| *tatC*-mut-F | 5’-ATGAGTCAGGGTGAAACCCAACATACCCC-3’ |
| *tatC*-mut-R | 5’-TCAGCTCCCCTCAGCGATCGGCTCG-3’ |
