# Supplementary material for: The temperature-dependent expression of type II secretion system controls extracellular product secretion and virulence in mesophilic Aeromonas salmonida SRW-OG1
Source: Front Cell Infect Microbiol. 2022 Aug 1;12:945000. doi: 10.3389/fcimb.2022.945000 (PMC9376225; doi:10.3389/fcimb.2022.945000)
Supplement: Supplementary file 2 [file Table_2.docx]

Table S2. Primer sequences used to validate gene knockout

| Target gene | Sequences (5’–3’) ^a^ |
| --- | --- |
| *tatA*-jd-F | 5’-ATGGGTGGTATCAGTATTTGGCA-3’ |
| *tatA*-jd-R | 5’-TTAAGCCTGATCTTTGTCTTTCTGC-3’ |
| *tatB*-jd-F | 5’-ATGTTCGATATCGGTTTCTGGG-3’ |
| *tatB*-jd-R | 5’-TCATGGCTTCACCTCCTCTTTT-3’ |
| *tatC*-jd-F | 5’-ATGAGTCAGGCCGAACAACCT-3’ |
| *tatC*-jd-R | 5’-TCAGCTCCCCTCTTCCGTTT-3’ |
