# Supplementary material for: The temperature-dependent expression of type II secretion system controls extracellular product secretion and virulence in mesophilic Aeromonas salmonida SRW-OG1
Source: Front Cell Infect Microbiol. 2022 Aug 1;12:945000. doi: 10.3389/fcimb.2022.945000 (PMC9376225; doi:10.3389/fcimb.2022.945000)
Supplement: Supplementary file 3 [file Table_3.docx]

Table S3. Primers for validating enzyme encoding genes by qRT-PCR

| Primers | Sequences (5’–3’) ^a^ |
| --- | --- |
| 16s-F | 5’-GGGGAGTACGGTCGCAAGAT-3’ |
| 16s-R | 5’-CGCTGGCAAACAAGGATAAGG-3’ |
| *tatA*-F | 5’-GTAGTCTTGCTGTTCGGC-3’ |
| *tatA*-R | 5’- TTGTCTTTCTGCTTGGCG -3’ |
| *tatB*-F | 5’-TACCATCCATCCTGCCCC-3’ |
| *tatB*-R | 5’-GCTCTGAACGGTTGTCGC-3’ |
| *tatC*-F | 5’-GCCACCATACTGCTCTGC-3’ |
| *tatC*-R | 5’-TCCTGTTGCTCCTCATCC-3’ |
| *cyoE*-F | GCCAAACACCACATCACCC |
| *cyoE*-R | CCACCGACATCATCACGCT |
| *ahh1*-F | GGCGGGGTTGAAGTGAA |
| *ahh1*-R | TGGCGGTCTTGGAGGAG |
| *lipA*-F | CCATCTGCACCCGTCGTT |
| *lipA*-R | CTCTTCGTTGGTCTCGCC |
| *lipB*-F | TACCCTGGCAAAAAGCGA |
| *lipB*-R | CGTGAAACGAGCAACCCT |
| *pulA*-F | CGACATGAAGGTGGTTTT |
| *pulA*-R | CGCTGATGCTCTGGGACT |
| *HED66_RS01350*-F | CCCCCGAGATAAAACAGG |
| *HED66_RS01350*-R | TTTGAACAGCACGAAGCA |
| *HED66_RS19960*-F | ACGAGTGCGAGAATGTCC |
| *HED66_RS19960*-R | TGCTGCTGAATCGGTTAC |
| *aspA*-F | CAACGACGCCTACCCTA |
| *aspA*-R | CGTGGAACTCCTGACCC |
| *fabD*-F | GTGGCAACAGCAGGGAGG |
| *fabD*-R | GAGATGGCACAGAGACCG |
| *gpsA*-F | GACGGGCTGGGGTTTGGT |
| *gpsA*-R | GCGGCTTCTTTCGGGTTC |
