# Supplementary material for: The temperature-dependent expression of type II secretion system controls extracellular product secretion and virulence in mesophilic Aeromonas salmonida SRW-OG1
Source: Front Cell Infect Microbiol. 2022 Aug 1;12:945000. doi: 10.3389/fcimb.2022.945000 (PMC9376225; doi:10.3389/fcimb.2022.945000)
Supplement: Supplementary file 4 [file Table_4.docx]

Table S4. Determination of enzymatic activity of extracellular products of *A. salmonicida* when cultured at different temperatures

| Species | Strains | Temp/℃ | Extraclelular protein bioactivities | | | | | | |
| --- | --- | --- | --- | --- | --- | --- | --- | --- | --- |
|  |  |  | Caseinase | Amylase | Lipase | gelatinase | Urease | Hemolysis | lecithinase |
| *Aeromonas salmonicid* | SRW-OG1 | 18℃ | ＋ | ＋ | ＋ | － | － | ＋ | ＋ |
|  | SRW-OG1 | 28℃ | ＋ | ＋ | ＋ | － | － | ＋ | ＋ |
|  | SRW-OG1 | 37℃ | ＋ | ＋ | ＋ | － | － | － | ＋ |
|  | ∆*tatA* | 28℃ | ＋ | ＋ | ＋ | － | － | ＋ | ＋ |
|  | ∆*tatB* | 28℃ | ＋ | ＋ | ＋ | － | － | ＋ | ＋ |
|  | ∆*tatC* | 28℃ | ＋ | ＋ | ＋ | － | － | ＋ | ＋ |
